# Association of the interaction between daily step counts and frailty with disability in older adults

**DOI:** 10.1007/s11357-024-01471-y

**Published:** 2024-12-21

**Authors:** Daiki Watanabe, Tsukasa Yoshida, Yuya Watanabe, Yosuke Yamada, Motohiko Miyachi, Misaka Kimura

**Affiliations:** 1 Faculty of Sport Sciences, Waseda University, 2-579-15 Mikajima, Tokorozawa City, Saitama, 359-1192 Japan; 2 National Institute of Health and Nutrition, National Institutes of Biomedical Innovation, Health and Nutrition, 3-17 Senrioka-Shimmachi, Settsu City, Osaka, 566-0002 Japan; 3 Institute for Active Health, Kyoto University of Advanced Science, 1-1 Nanjo Otani, Sogabe-cho, Kameoka City, Kyoto, 621-8555 Japan; 4 Senior Citizen’s Welfare Section, Kameoka City Government, 8 Nonogami, Yasu-machi, Kameoka City, Kyoto, 621-8501 Japan; 5 National Institute of Biomedical Innovation, National Institutes of Biomedical Innovation, Health and Nutrition, 7-6-8 Saito-Asagi, Ibaraki City, Osaka, 567-0085 Japan; 6 Faculty of Sport Study, Biwako Seikei Sport College, 1204 Kitahira, Otsu City, Shiga 520-0503 Japan; 7 Sports and Health Sciences, Graduate School of Biomedical Engineering, Tohoku University, 2-1 Seiryo-machi, Aoba-ku, Sendai City, Miyagi 980-8575 Japan; 8 Laboratory of Applied Health Sciences, Kyoto Prefectural University of Medicine, 465 Kajii-cho, Kamigyo-ku, Kyoto City, Kyoto, 602-8566 Japan

**Keywords:** Physical activity, Frailty, Long-term care insurance, Kihon Checklist, Dose–response relationships

## Abstract

The differences in the association of objectively measured physical activity with disability between frail and non-frail older adults remain unclear. We (1) evaluated the dose-dependent relationship between daily steps and disability in older adults with and without frailty and (2) examined the interaction between steps and frailty status in relation to the risk of disability. This prospective study used data from 4065 adults aged ≥ 65 years from the Kyoto-Kameoka Study, Japan. The mean daily step count obtained using triaxial accelerometers across ≥ 4 days was recorded. Frailty was evaluated using the validated Kihon Checklist. Disability was identified using the long-term care insurance system’s nationally unified database, with data collected until November 30, 2016. Overall, 385 disabilities were recorded during a median follow-up period of 3.32 years (12,855 person-years). After adjusting for confounders, an inverse association was observed between daily step count and disability risk. The disability risk plateaued at 5,000–7,000 steps/day in non-frail people, whereas step counts showed an almost linear inverse relationship with disability risk in frail people. Low step counts (< 5,000 steps) in frail people were more strongly associated with disability risk than were high step counts (≥ 5,000 steps) in non-frail people. The additive interaction between steps and frailty was associated with the relative excess risk of disability in frail people with low step counts (p for interaction = 0.015). The relationship between daily steps and disability differs between older adults with and without frailty, and the adverse effects of frailty on disability risk depend on physical activity.

**Supplementary Information:**

The online version contains supplementary material available at 10.1007/s11357-024-01471-y.

## Introduction

Frailty is characterized by impaired integrity of multiple physiological systems due to the loss of health equilibrium resulting from stress responses related to multidimensional risk factors, such as physical and psychosocial abilities [1, 2]. It is closely associated with disability [3] and mortality [4, 5]. Although prolongation of lifespan is important for frail older adults, appropriate care to ensure the preservation of the capacity to live independently and function well during late life is of even greater significance [6].

Physical inactivity is a major modifiable cause of adverse health outcomes [7, 8], and its prevalence has been increasing in high-income countries [9]. Since the daily step count is a simple and easily comprehensible objective measure of physical activity (PA), it is an effective tool for setting PA goals and motivating individuals to increase PA [10, 11]. Step counts have been shown to vary greatly across countries and territories [12], as well as among individuals with different frailty statuses [13, 14]. Therefore, appropriate step count goals that older adults with and without frailty can achieve daily are essential for maintaining health status.

Several prospective cohort studies have indicated that objectively measured PA is inversely associated with disability in older adults [15, 16]. The Lifestyle Interventions and Independence for Elders (LIFE) study reported that interventions to increase PA, such as walking with a goal of 150 min/week, are more effective in preventing mobility-related disability incidents in people with poor lower limb function than in those with better lower limb function [17]. However, the differences in the association between PA and disability in older adults in relation to the presence of frailty and the impact of the interaction between PA and frailty on the risk of disability remain unclear [3, 18]. Therefore, this study aimed (1) to evaluate the dose-dependent relationship between daily step counts and disability in older adults with and without frailty, and (2) to examine the interaction between step counts and frailty status in relation to the risk of disability. Based on the abovementioned studies [17], we hypothesized that (1) the benefits of higher PA in reducing disability risk in frail individuals were greater than those in non-frail individuals, and (2) the combination of low PA and frailty was strongly associated with the risk of disability through the interaction of these factors.

## Methods

### Study population and assessment of baseline characteristics

**Fig. 1:**
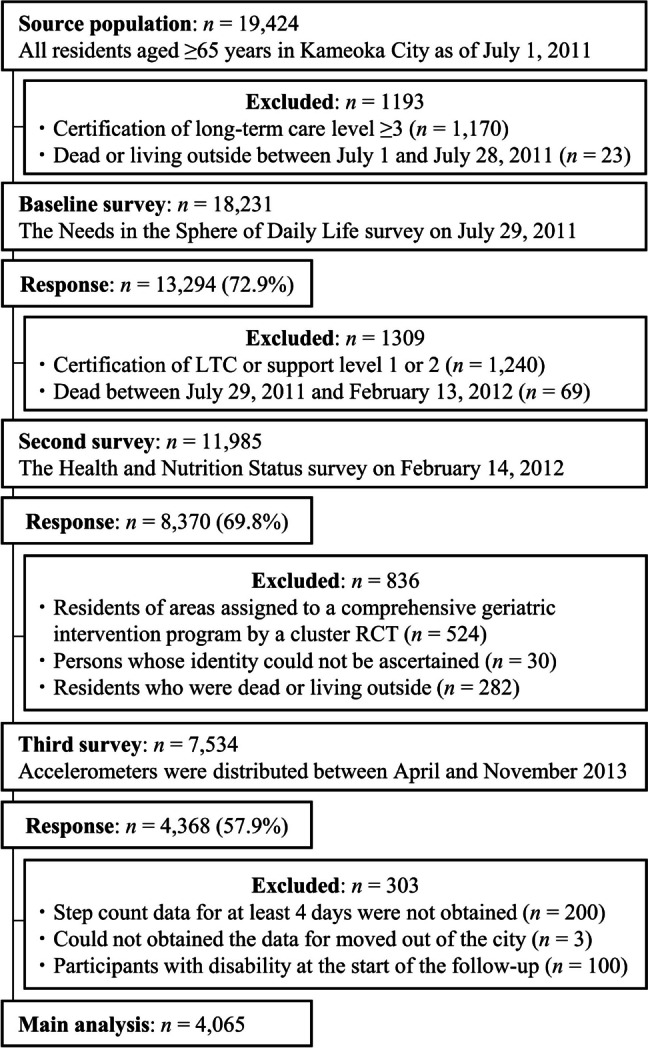
Participant flow diagram for the analysis of daily step counts and disability in the Kyoto-Kameoka study. RCT, randomized controlled trial, LTC, long-term care

The Kyoto-Kameoka Study is a population-based prospective cohort study of adults aged ≥ 65 years living in Kameoka, Kyoto, Japan. Details of the study are explained elsewhere [5, 13, 14, 19–22]. Briefly, this study was conducted among all residents of Kameoka City aged ≥ 65 years as of July 1, 2011, with 13,294 residents providing valid responses for the baseline survey (Fig. 1). After the first baseline survey, the Health and Nutrition Status Survey (pre-trial [second] survey) was conducted by postal mail in the district on February 14, 2012, obtaining responses from 8370 residents (response rate = 69.8%). Among these, residents who were assigned to the comprehensive geriatric intervention program as part of the Kyoto-Kameoka cluster randomized controlled trial (RCT; *n* = 524), residents who could not be identified (*n* = 30), and those who moved out of the city or died (*n* = 282) were excluded. Accelerometers were distributed to the remaining 7534 people from April to November 2013 (third survey). Step count measurements were performed on 4368 people (response rate = 57.9%) for at least one day. All participants provided informed consent when responding to the mail survey. This study was conducted according to the guidelines laid down in the 1964 Declaration of Helsinki, and all procedures involving research participants were approved by the Research Ethics Committee of Kyoto Prefectural University of Medicine (RBMR-E-363), the National Institutes of Biomedical Innovation, Health and Nutrition (NIBIOHN-76–2), and Kyoto University of Advanced Science (No. 20–1). All participants provided informed consent when responding to the mail survey. This study was reported in accordance with the Strengthening the Reporting of Observational Studies in Epidemiology (STROBE) guidelines [23].

Among the participants included at baseline (*n* = 4368), we excluded individuals with missing step count data, as evidenced by the lack of appropriate accelerometer use (*n* = 200) [13, 14], those with an unknown date of moving away from the community (*n* = 3), and those with disabilities at the start of the follow-up period (*n* = 100). Ultimately, 4065 participants were included in this study.

### Assessment of daily step counts

The step count was measured as an objective PA index using a triaxial accelerometer (EW-NK52; Panasonic Co., Ltd., Osaka, Japan) as described previously [13, 14]. From April 1 to November 15, 2013, we mailed residents an accelerometer along with printed instructions on its use and asked them to wear it for 10 or more days. Participants were instructed to wear the accelerometer on their waists from when they woke up until they went to bed, but not during sleeping, bathing, and swimming; otherwise, they were asked to continue with their routine tasks as usual. The daily step count was determined using the manufacturer’s step algorithm. Since the wear time data for the accelerometer were not available, we excluded individuals with lower wear times [13, 14] by removing step count data points lower than the first percentile (499 steps for males and 653 for females) in older adults aged 60–69 years, as reported in the National Health and Nutrition Surveys Japan (NHNS-J) [24]. To calculate the daily step count, we summed the steps surveyed for ≥ 4 days (including one holiday) [13] and divided this by the number of days to obtain the average daily step count. Individual male and female measurements estimated based on a 4-day step survey had a correlation coefficient (r) of 0.90 with respect to the “true” average step count, suggesting that a 4-day accelerometer study was sufficient to reflect the individuals’ habitual step counts [13]. We needed 3658 participants to estimate the “true” mean step count of the group within a 95% confidence interval (CI) and error margin of 2.5% [13]. Therefore, the sample size and number of assessment days for step counts in this study were sufficiently large.

### Definition of frailty

Frailty was assessed using the validated self-administered Kihon Checklist (KCL) consisting of 25 questions [5, 20]. The KCL evaluates frailty from multiple aspects, including physical, social, and cognitive factors (comprehensive frailty). Frailty was defined by at least seven positive responses in the list of 25 items [5, 20]. A prospective cohort study showed that KCL scores were associated with the risk of death in older adults during a 5-year study period [5].

### Outcomes

Disability incidence was identified using the long-term care insurance system’s nationally unified database in Japan [21, 25]. Disability (≥ support level 1), as defined by this system, is a condition in which some assistance is required for instrumental daily living activities. Candidates with disabilities underwent an in-person assessment of everyday functioning by officials (dispatched by the local government) using a 74-item questionnaire based on the activities of daily living. Based on the questionnaire results and physician opinions, the candidates’ functional disability levels were determined by the Long-Term Care Insurance Certification Committee, which consisted of academic experts in healthcare and welfare. This outcome information was provided by the officials of Kameoka City Hall and collected until November 30, 2016. Residents whose records were administratively removed or who moved out of the municipality were excluded from the study.

### Statistical analysis

Daily step counts were classified into quartiles. Descriptive statistics for continuous and categorical variables are shown as mean and standard deviation and number and percentage, respectively. Missing covariates created missing indicator variables. Details regarding the assessment of these covariates and statistical analyses are provided in the Supplementary Methods.

We calculated each participant’s person-years of follow-up from the obtained date of daily steps to the date of functional disability, relocation from the study area, or end of follow-up, whichever occurred first. The rate of disability for each daily step count quartile was shown as the number of events per 1,000 person-years. We used the multivariable sub-distribution hazard model approach proposed by Fine and Gray [26], which included baseline covariates to adjust for confounding factors associated with step count and disability. The results of these analyses were presented as sub-distribution hazard ratios (SHRs) and 95% CIs. The SHRs were calculated using the first quartile as the reference. The *p*-value of the linear trend was calculated by considering step count exposure as a continuous variable. We also used a restricted cubic spline model with three knots (5th, 50th, and 95th percentiles), based on the distribution of steps, to evaluate the curvilinearity of the relationship between the step count and disability [13, 14]. These results are presented as SHRs and 95% CIs, with the SHR calculated using the average value of the first step count quartile as the reference value. Because the data were sparse, we truncated the analysis at 14,000 steps/day (99.5% of the distribution) [13, 14]. The statistical significance of nonlinearity was assessed using a Wald test, which compared the likelihood ratio of the spline model with the linear model, and *p*-values < 0.05 indicated a statistically significant nonlinear relationship between the exposure and outcome [14].

We estimated the propensity scores for assignment into the daily step count quartiles using a multivariable logistic regression model that included the variables in Model 2 and created multivariable-adjusted Nelson–Aalen cumulative hazard curves using inverse probability weighting methods.

To assess the interaction between daily steps and frailty with disability, the participants were classified into the following four groups: high steps (≥ 5,000 steps/day)/non-frail group, *n* = 1,007; high steps (≥ 5,000 steps/day)/frail, *n* = 206; low steps (< 5,000 steps/day)/non-frail group, *n* = 2,045; and low steps (< 5,000 steps/day)/frail group, *n* = 807. The interaction between an outcome and exposure is best assessed using both additive and multiplicative interaction measurements [27]. Therefore, we have calculated both the additive interaction (relative excess risk due to interaction [RERI]) and multiplicative interaction. The RERI was calculated using step count and frailty as categorical variables [19]. The RERI and 95% CI were calculated using the “nlcom” command in STATA. The following equation was used: RERI = (SHR [low steps/frail] − 1) − (SHR [low steps/non-frail] + SHR [high steps/frail] − 2). Values are presented as RERI (95% CI), and were considered significant (*p* < 0.05) if the 95% CI of the RERI was not above 0.

We performed sensitivity analyses using the following three methods: 1) to eliminate the possibility of reverse causal relationships, we excluded disability events (64 men and 50 women) recorded in the first year of follow-up; 2) we performed a similar analysis using a dataset in which missing values for covariates were supplemented with multiple imputations; and 3) we adjusted for each of the chronic diseases but not for the number of chronic diseases. Single imputation of missing values, such as using the mean of the observed values and a missing category indicator, usually causes standard errors to be too small because it fails to account for the uncertainty associated with the missing values [28]. This analysis comprised pooled results from 20 datasets created with random numbers using the multiple-imputation method to supplement missing covariate values with the “mi estimate” command in STATA. All missing values were presumed to be random.

Multivariate analysis, which avoids multicollinearity, was conducted by modeling the potential confounders reported in previous studies [13, 14, 19, 29–31]. The multivariate analysis was verified using two models. Model 1 included adjustments for age, sex, population density, and the season in which step count assessments were performed. Model 2 included adjustments for all variables in Model 1 along with body mass index, smoking status, alcohol consumption, living alone, educational attainment, socioeconomic status, sleep duration, sitting time, denture use, medication use, number of chronic diseases, and frailty.

A two-tailed probability of less than 5% was considered significant for all statistical analyses. Statistical analyses were performed using STATA MP, Version 15.0 (StataCorp LP, College Station, TX, USA).

## Results

Table 1 shows the baseline participant characteristics grouped by daily step count quartiles. The mean daily step count (standard deviation) for the entire population was 4232 (2402). Participants with higher step counts included larger numbers of people not taking medications, men, younger individuals, and alcohol consumers, and fewer frail individuals. Furthermore, this study included fewer participants with frailty and fewer disability events than the baseline (pre-trial) survey (Supplemental Table 1).

**Table 1:** Baseline characteristics of the study participants by quartile of daily step count

	Total (*n* = 4065)		Quartile of the daily step count							
			Q1 (*n* = 1018)		Q2 (*n* = 1015)		Q3 (*n* = 1016)		Q4 (*n* = 1016)	
Age [years] ^a^	72.2	(5.1)	74.4	(6.0)	72.5	(5.1)	71.3	(4.7)	70.3	(4.3)
Women [*n* (%)] ^b^	1972	(48.5)	529	(52.0)	553	(54.5)	510	(50.2)	380	(37.4)
PD ≥ 1000 people/km^2^ [*n* (%)] ^b^	1985	(48.8)	499	(49.0)	533	(52.5)	491	(49.3)	462	(45.5)
Body mass index [kg/m^2^] ^a^	22.7	(3.1)	22.8	(3.5)	22.8	(3.4)	22.6	(2.8)	22.4	(2.6)
Current smoker [*n* (%)] ^b^	408	(10.0)	125	(12.3)	92	(9.1)	101	(9.9)	90	(8.9)
Alcohol drinker [*n* (%)] ^b^	2745	(67.5)	640	(62.9)	649	(63.9)	699	(68.8)	757	(74.5)
Living alone [*n* (%)] ^b^	455	(11.2)	112	(11.0)	132	(13.0)	123	(12.1)	88	(8.7)
Education ≥ 13 y [*n* (%)] ^b^	915	(22.5)	201	(19.7)	212	(20.9)	236	(23.2)	266	(26.2)
HSES [*n* (%)] ^b^	1396	(34.3)	324	(31.8)	363	(35.8)	351	(34.6)	358	(35.2)
Sleep time [min/day] ^a^	400	(78)	409	(87)	397	(76)	398	(75)	396	(73)
Sitting time [min/day] ^a^	300	(197)	347	(218)	302	(199)	285	(187)	266	(181)
Denture use [*n* (%)] ^b^	2263	(55.7)	606	(59.5)	573	(56.5)	567	(55.8)	517	(50.9)
No medication [*n* (%)] ^b^	954	(23.5)	194	(19.1)	207	(20.4)	248	(24.4)	305	(30.0)
Hypertension [*n* (%)] ^b^	1474	(36.3)	425	(41.8)	364	(35.9)	337	(33.2)	348	(34.3)
Stroke [*n* (%)] ^b^	118	(2.9)	33	(3.2)	31	(3.1)	22	(2.1)	32	(3.2)
Heart disease [*n* (%)] ^b^	446	(11.0)	150	(14.7)	118	(11.6)	80	(7.9)	98	(9.7)
Diabetes [*n* (%)] ^b^	384	(9.5)	107	(10.5)	87	(8.6)	90	(8.9)	100	(9.8)
Hyperlipidemia [*n* (%)] ^b^	444	(10.9)	109	(10.7)	129	(12.7)	119	(11.7)	87	(8.6)
Digestive disease [*n* (%)] ^b^	337	(8.3)	101	(9.9)	85	(8.4)	84	(8.3)	67	(6.6)
Respiratory disease [*n* (%)] ^b^	168	(4.1)	55	(5.4)	45	(4.4)	38	(3.7)	30	(3.0)
Urological disease [*n* (%)] ^b^	265	(6.5)	66	(6.5)	64	(6.3)	53	(5.2)	82	(8.1)
Cancer [*n* (%)] ^b^	114	(2.8)	35	(3.4)	34	(3.4)	29	(2.9)	16	(1.6)
No. of chronic diseases ^a,c^	0.92	(0.95)	1.06	(1.03)	0.94	(0.94)	0.84	(0.91)	0.85	(0.91)
Frailty [*n* (%)] ^b^	1013	(24.9)	362	(35.6)	268	(26.4)	215	(21.2)	168	(16.5)
Daily step count [steps/day] ^a^	4232	(2402)	1815	(427)	3071	(355)	4495	(489)	7550	(2064)

*HSES* High socioeconomic status; *PD* Population density; *Q* Quartile. Number of missing values for variables are as follows: body mass index (*n* = 6; 0.1%), smoking status (*n* = 145; 3.6%), alcohol drinker (*n* = 122; 3.0%), family structure (*n* = 259; 6.4%), educational attainment (*n* = 392; 9.6%), socioeconomic status (*n* = 167; 4.1%), sleep duration (*n* = 139; 3.4%), sitting time (*n* = 401; 9.9%), denture use (*n* = 106; 2.6%), medications (*n* = 279; 6.9%), and frailty status (*n* = 485; 11.9%). Body mass index was calculated as body weight (kg) divided by height squared (m^2^). Q1 to Q4 consist of daily step counts of < 2477, 2478–3691, 3692–5419, and ≥ 5420 steps, respectively

^a^ Continuous values are shown as mean (standard deviation)

^b^ Categorical values are shown as number (percentage)

^c^ From the data obtained on disease status (including the presence of hypertension, stroke, heart disease, diabetes, hyperlipidemia, digestive disease, respiratory disease, urological diseases, and cancer), the comorbidity scores were summed to obtain a total score ranging from 0 (no comorbidity) to 9 (poor status)

**Fig. 2:**
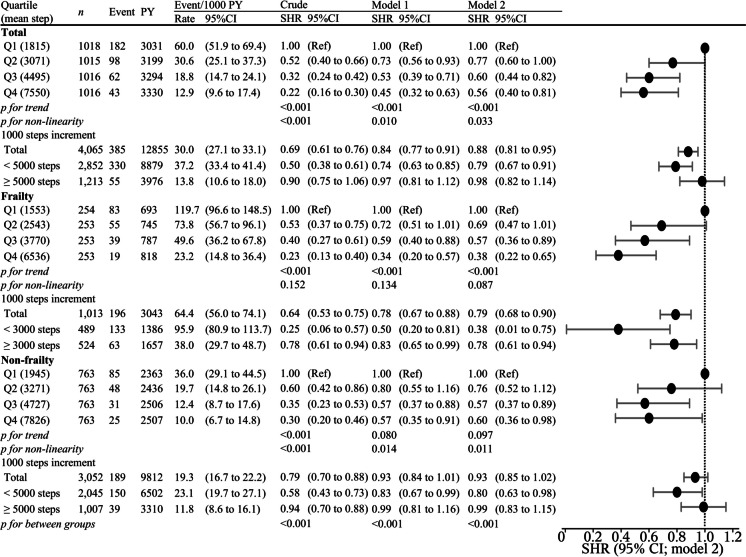
Association between daily step counts and disability calculated using the multivariable sub-distribution hazard model among older adults. Q1 through Q4 include daily step counts of < 2477, 2478–3691, 3692–5419, and ≥ 5420 steps, respectively, in the total participants; < 2079, 2080–3079, 3085–4565, and ≥ 4587 steps, respectively, in frail individuals; and < 2643, 2644–3937, 3938–5682, and ≥ 5683 steps, respectively, in non-frail individuals. Model 1 adjusted for age (continuous), sex (women or men), population density (≥ 1000 or < 1000 people/km^2^), and the step count assessment season (spring, summer, or autumn); Model 2 adjusted for all variables in Model 1 and body mass index (< 18.5, 18.5–24.9, 25–29.9, ≥ 30 kg/m^2^, or missing), smoking status (never smoked, past smoker, current smoker, or missing), alcohol drinking (never drank, past drinker, current drinker, or missing), living alone (living alone, living with others, or missing), educational attainment (< 9, 10–12, ≥ 13 years, or missing), socioeconomic status (high, low, or missing), sleep duration (< 360, 360 to < 420, 420 to < 480, ≥ 480 min/day, or missing), sitting time (< 5, 5 to < 7, 7 to < 9, ≥ 9 h/day, or missing), denture use (yes, no, or missing), medication use (none, 1, 2, 3, 4, ≥ 5, or missing), number of chronic diseases (continuous), and frailty (yes or no). CI, confidence interval; SHR, subdistribution hazard ratio; PY, person-years; Q, quartile; Ref, reference

**Fig. 3:**
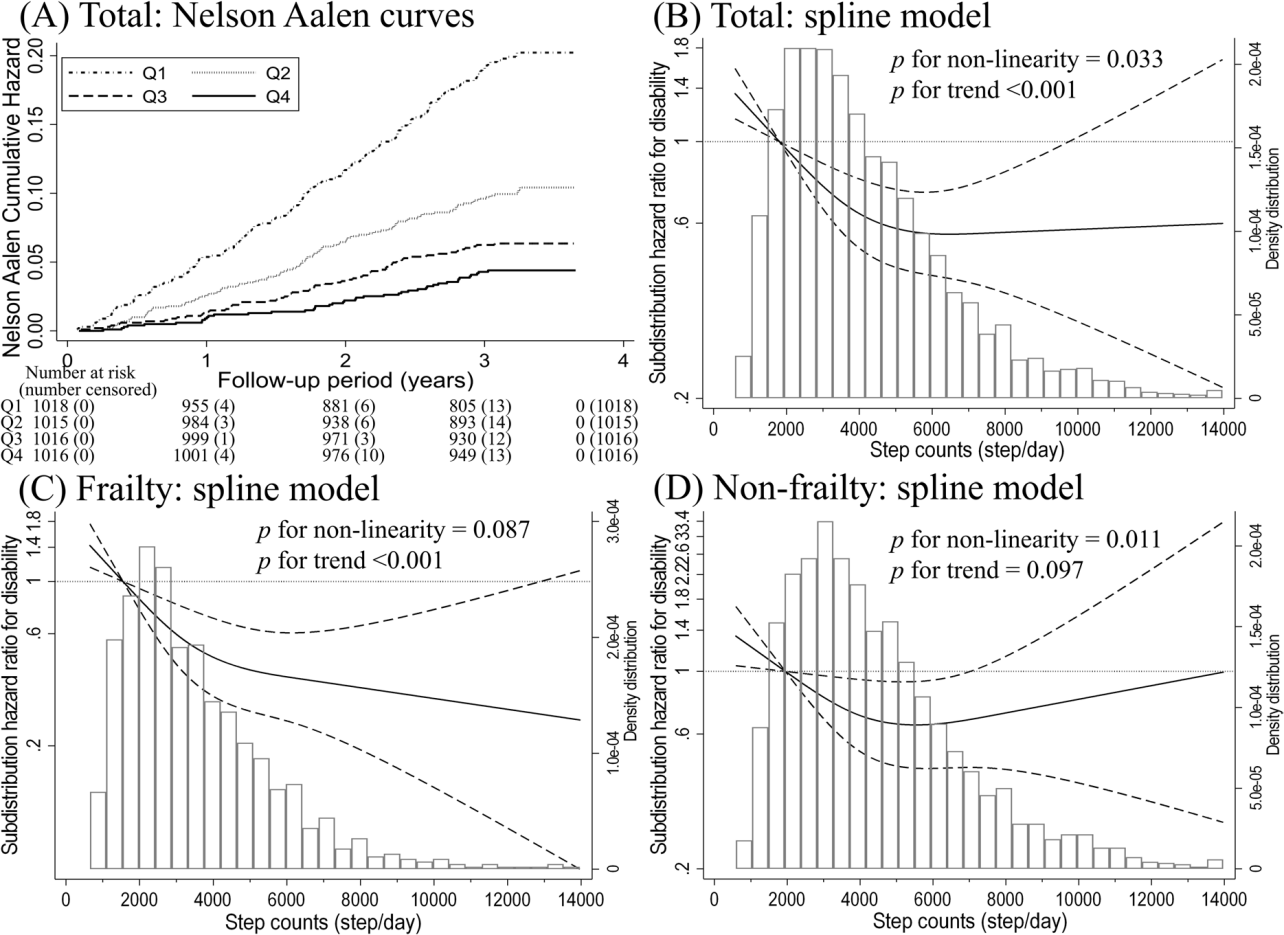
Nelson–Aalen cumulative hazard curves and restricted cubic spline regression model between daily step count and risk of disability among older adults. (**A**) Multivariable adjusted Nelson–Aalen cumulative hazard curves using inverse probability weighting according to quartiles (Qs) of daily step counts and (**B-D**) restricted cubic spline regression model. Solid lines represent subdistribution hazard ratios, dashed lines represent 95% confidence intervals (CI), and the hazard ratios based on (**B**) 1815 steps/day in the total participants (*n* = 4052), (**C**) 1553 steps/day in frail individuals (*n* = 1011), and (**D**) 1945 steps/day in non-frail individuals (*n* = 3041) as the reference (mean step counts for the first quartile value) were calculated. We estimated that *p* < 0.05 when the 95% CI of the subdistribution hazard ratio did not exceed 1.00 and *p* ≥ 0.05 when the 95% CI of the subdistribution hazard ratio exceeded 1.00. The adjustment factors were age, sex, population density, season of wear, body mass index, smoking status, alcohol consumption, family structure, educational level, economic status, sleep duration, sitting time, denture use, medication use, chronic disease count, and/or frailty status

Figures 2 and 3A show the relationship between daily step counts and disability. The median follow-up period for all participants was 3.32 years (interquartile range: 3.21–3.46 years). In total, 385 (9.5%) disabilities were recorded during the follow-up period (12,855 person-years). After adjusting for confounders, an inverse association was observed between the daily step count and risk of disability. Similar results were obtained in the sensitivity analysis (Supplemental Tables 2–4). The daily step count at which the SHR for disability plateaued among all participants was approximately 5000–6000 steps/day (*p* for nonlinearity = 0.033; Fig. 3B). The spline analysis model fit the data better than the linear regression analysis (Akaike Information Criterion: 5877 vs. 5880).

Figures 2 and 3C, D show the relationship between step count and disability in individuals with and without frailty. The prevalence of frailty in this population was 24.9% (95% CI: 23.6–26.3). In the spline models, the decline in the SHR for disability with the daily step count among non-frail individuals plateaued at approximately 5,000–7,000 steps/day (p for nonlinearity < 0.001; Fig. 3D). However, in frail individuals, daily step counts showed a strong inverse relationship with mortality up to approximately 4,000–5,000 steps/day and a moderate, dose-dependent, inverse relationship up to approximately 14,000 steps/day (p for nonlinearity < 0.001; Fig. 3C).

Table 2 presents the relationships between step counts/frailty status and the risk of disability. The risk of disability was higher in the low steps/frailty group than in other groups (low steps/frail group: SHR, 2.39 [95% CI: 1.62–3.53]). The additive interaction between daily step counts and frailty accounted for the excess risk of disability in the low steps/frail group (RERI, 0.79 [95% CI: 0.11–1.47], *p* = 0.015). However, the multiplicative interaction was not significant. Similar results were obtained in the sensitivity analysis (Supplemental Tables 5–7).

**Table 2:** Association of the interaction between daily step counts and frailty with disability in older Japanese adults

	*n*	Event	PY	Event/1000 PY		Crude		Model 1^a^		Model 2^b^	
				Rate	95% CI	SHR	95% CI	SHR	95% CI	SHR	95% CI
SC × Frail											
HSC/Non-frail	1007	39	3310	11.8	(8.6 to 16.1)	1.00	(Ref)	1.00	(Ref)	1.00	(Ref)
HSC/Frail	206	16	666	24.0	(14.7 to 39.2)	2.04	(1.14 to 3.63)	1.35	(0.94 to 1.93)	1.25	(0.86 to 1.82)
LSC/Non-frail	2045	150	6502	23.1	(19.7 to 27.1)	1.93	(1.36 to 2.75)	1.64	(0.91 to 2.96)	1.35	(0.72 to 2.51)
LSC/Frail	807	180	2377	75.7	(65.4 to 87.7)	6.38	(4.52 to 9.02)	3.12	(2.17 to 4.49)	2.39	(1.62 to 3.53)
Interaction											
RERI^c^						3.41	(1.87 to 4.95)	1.13	(0.35 to 1.91)	0.79	(0.11 to 1.47)
*p*-value						< 0.001		0.001		0.015	
Multiplicative						1.62	(0.87 to 3.00)	1.41	(0.75 to 2.65)	1.41	(0.74 to 2.70)
*p*-value						0.125		0.285		0.301	
Step counts											
≥ 5000 steps	1213	55	3976	13.8	(10.6 to 18.0)	1.00	(Ref)	1.00	(Ref)	1.00	(Ref)
< 5000 steps	2852	330	8879	37.2	(33.4 to 41.4)	2.66	(2.00 to 3.53)	1.67	(1.24 to 2.23)	1.41	(1.04 to 1.91)
Frailty status											
Non-frail	3052	189	9812	19.3	(16.7 to 22.2)	1.00	(Ref)	1.00	(Ref)	1.00	(Ref)
Frail	1013	196	3043	64.4	(56.0 to 74.1)	3.35	(2.74 to 4.09)	2.20	(1.78 to 2.71)	1.82	(1.46 to 2.28)

*CI* Confidence interval; *SHR* Subdistribution hazard ratio; *HSC* High step counts; *LSC* Low step counts; *PY* Person years; *Ref* Reference; *RERI* Relative Excess Risk due to Interaction; *SC* Step counts

^a^ Model 1: Adjusted for age, sex, population density, and season of wear

^b^ Model 2: Adjusted for Model 1 variables and body mass index, smoking status, alcohol consumption status, family structure, educational attainment, economic status, sleep duration, sitting time, denture use, medication use, and number of chronic diseases

^c^ The additive interaction was calculated as the RERI using the following equation: RERI = (SHR [LSC/Frail] − 1) − (SHR [LSC/Non-frail] + SHR [HSC/Frail] − 2). The values are shown as RERI (95% CI). It is significant (*p* < 0.05) if the 95% CI of the RERI is not above 0

## Discussion

The risk of disability plateaued at 5,000–7,000 steps/day in non-frail individuals, whereas the association between the number of steps and the risk of disability was almost linearly inverse in frail people; the additive interaction between steps and frailty was associated with the relative excess risk of disability. To our knowledge, this is the first study to demonstrate that the association between objectively assessed PA and disability differs between older adults with and without frailty, and to examine the interaction between PA and frailty status in relation to the risk of disability.

Our results indicate that an increase in step count may offer a greater benefit in terms of reduced disability risk in frail individuals than in non-frail individuals. Similar results have been observed for the association between PA and mortality in previous studies [29–31]. An intervention study to increase PA for 2 years also showed that those with higher levels of frailty benefited more from the preventive effect of the intervention on mortality risk [3]. Although intervention studies can help interpret causal relationships between outcomes and exposure factors, they cannot evaluate the optimal target value of PA for disability prevention. Therefore, although further well-designed prospective cohort studies are needed to verify this finding, quantifying the dose–response relationship between the number of steps and disability stratified by those with and without frailty may be useful for setting targets for future PA guidelines and developing public health policies.

Our findings revealed an additive interaction between step count/frailty status and the risk of disability, but no significant multiplicative interaction was found. To our knowledge, a significant association of the additive interaction between PA and frailty with disability has not yet been reported [3, 18]. Additive interactions are more useful than are multiplicative interactions in assessing public health importance because it provides more insight into which subgroups might be best served by a treatment or intervention [27]. In addition, tests for additive interaction are sometimes more powerful than are tests for multiplicative interaction; therefore, for the purposes of discovery and detection, the additive scale may be preferred as well [27]. The LIFE study assessed mobility-related disability based on the ability to walk 400 m within 15 min [3, 17, 18], but our study instead used a comprehensive disability assessment tool with multidimensional domains. In addition, the LIFE study reported a trend of interaction between PA and lower limb function assessed by the Short Physical Performance Battery with disability [17], but no such associations were observed with the frailty index [3, 18]. Because the association of the interaction between PA and frailty with disability may depend on the method of frailty assessment, these results indicate that PA interventions should be personalized on the basis of the relevant aspects of frailty.

Several previous studies have reported sitting time as a factor that interacts with PA to influence health outcomes [19, 32]. Previous studies have indicated that individuals with high levels of sedentary behavior might benefit more in terms of a lower mortality risk from increasing PA than might those with low levels of sedentary behavior [33, 34]. Our study population consisted of older adults from the general population who experience longer sitting times and perform less PA [19] in comparison with older adults in previous studies, which may have made the interaction between PA and frailty easier to confirm. However, our results did not substantially change after adjusting for sitting time, indicating that this effect may be weak. Therefore, further research is needed because the adverse effects of low PA in frail individuals may be attributable to factors other than the longer sitting time associated with frailty.

The detailed mechanism by which the interaction or differences in frailty status and PA benefit disability risk remains unclear, but some previous studies have suggested two possible reasons. First, the step count was inversely associated with the maintenance of skeletal muscle mass and the proportion of frailty. We reported that the step count was inversely associated with the proportion of individuals with frailty [13]. Higher step counts may be associated with improvements in frailty status, which is associated with a lower disability risk. A mail-based walking intervention study showed that the walking intervention group had higher serum levels of the anabolic hormones dehydroepiandrosterone and insulin-like growth factor and greater skeletal muscle mass than the control group did [35]. Previous network meta-analyses have reported that the optimal interventions for preventing the onset of frailty are PA [36] and resistance exercise [37], and these studies may support our findings. Second, individuals with high step counts tend to maintain meaningful social relationships [38]. Social relationships can affect health outcomes [39] because they provide information and emotional experiences that promote adaptive behavior or neuroendocrine responses to acute or chronic stressors [40]. Lockdowns in response to the coronavirus disease 2019 pandemic have been associated with higher levels of stress, such as mental health problems and loneliness, in older adults [41], suggesting that poor social relationships may be associated with the progression of frailty by disrupting physiological stress homeostasis.

The prevalence or degree of frailty has recently increased in most adult age groups in the US [1] and older adults in Japan [42]. Therefore, achievable goals to improve the PA of frail individuals are essential [10, 11]. Although 10,000 steps is a typical target daily step count in software programs, including wearable devices [14, 43], the distribution of our step count data suggests that this target is difficult for older adults to achieve. Our results indicated that frail individuals with high step counts (≥ 5,000 steps/day) and non-frail individuals with low step counts did not show a significantly higher disability risk than did non-frail individuals with high step counts, suggesting that a high step count partially offsets frailty-related disability risk. We previously indicated that the daily step count showed an inverse relationship with mortality at approximately ≥ 5,000 steps/day in frail individuals [14]. Therefore, a “5,000 steps/day” plan as the target daily step count to improve health status, including lifespan and disability, could be an achievable goal for many older adults, especially frail individuals.

The strength of the present study is that it examined the relationship between daily step count measured using an accelerometer and disability in a large cohort of older adults. A previous study reported that objectively measured PA was more strongly associated with health outcomes than self-reported measurements were [44]. However, the present study had some methodological limitations. First, the possibility of bias in the step count data measured by the participants could not be ruled out. The characteristics and disability risks of the participants included in this study, as well as the residents who completed the baseline (pre-trial) survey, differed. The number of daily steps may have been underestimated due to the inclusion of low-adherence days from the accelerometer, as this study could not obtain the wear time of the accelerometer. Nevertheless, the mean daily step counts obtained from this study were similar to the median step counts for older people aged 70–79 years, as reported in the NHNS-J in 2016 [24]. Second, this study had a relatively short observation period. Previous studies have indicated a stronger association between step counts and mortality in shorter follow-up studies [43, 45].

However, similar results were observed in a sensitivity analysis that excluded disability events occurring after 1 year of follow-up. Third, the number of steps was measured only at baseline, whereas the participants’ habitual step counts may have changed during the follow-up period. Nevertheless, a previous study of older Japanese people showed that the individual daily step ranking at baseline was well maintained for 8 years (Spearman’s rank correlation coefficient ≥ 0.6) [46]. Our study’s participants may also have had a stable individual number of steps during the follow-up period. Finally, our study was adjusted for several confounders; however, residual confounders, such as inflammatory markers, certain chronic diseases, and dietary intake, in the association between the number of steps and disability cannot be ruled out. Additionally, many covariates used in our study were assessed using self-report questionnaires, which may have included systematic errors such as recall and reporting bias.

## Conclusion

Our results revealed that the relationship between daily step count and disability differed between older adults with and without frailty. Further studies are needed to evaluate whether a stronger inverse relationship between the steps count and risk of disability exists in individuals with frailty than in those without.

## Supplementary Information

Below is the link to the electronic supplementary material.

Supplementary file1 (DOCX 75 KB)

## Data Availability

All data sharing and collaboration requests should be directed to the corresponding author (d2watanabe@nibiohn.go.jp), TY (t-yoshida@nibiohn.go.jp), and YY (yamaday@nibiohn.go.jp).
