# Supplementary material for: Association of the interaction between daily step counts and frailty with disability in older adults
Source: GeroScience. 2024 Dec 21;47(3):3377–90. doi: 10.1007/s11357-024-01471-y (PMC12181542; doi:10.1007/s11357-024-01471-y)
Supplement: Supplementary file 1 — Supplementary file1 (DOCX 75 KB) [file 11357_2024_1471_MOESM1_ESM.docx]

**Supporting information**

“Association of the interaction between daily step counts and frailty with disability in older adults”

**SUPPLEMENTARY TABLES**

**Supplementary Table 1.** Characteristics of participants in baseline and additional surveys and accelerometer study in the Kyoto-Kameoka Study

**Supplementary Table 2.** Results of sensitivity analysis for the relationship between daily step count and risk of disability after excluding participants with an event in the first year of follow-up

**Supplementary Table 3.** Results of sensitivity analysis for the relationship between daily step count and risk of disability using a multiple imputation method for missing values of covariates

**Supplementary Table 4.** Results of sensitivity analysis for the relationship between daily step count and risk of disability after adjusting for each of the chronic diseases

**Supplementary Table 5.** Results of sensitivity analysis for the association of the interaction between daily step count and frailty with disability after excluding participants with an event in the first year of follow-up

**Supplementary Table 6.** Results of sensitivity analysis for the association of the interaction between daily step count and frailty with disability using a multiple imputation method for missing values of covariates

**Supplementary Table 7.** Results of sensitivity analysis for the association of the interaction between daily step count and frailty with disability after adjusting for each of the chronic diseases

**Supplementary methods**

***Other covariates***

All covariates were obtained from questionnaire data from a baseline survey. We collected data on the following basic characteristics: smoking status (“Do you smoke?”: almost daily; sometimes; used to, but quit; never); drinking status (“Do you drink alcohol?”: almost daily, sometimes, almost never, never); sleep duration (minutes); living status (“What is your family structure?”: living alone, living with family, other); educational attainment (years); socioeconomic status (“Economically, how does your life feel currently?”: hard, somewhat hard, somewhat easy, easy); oral status (“Do you use dentures?”: yes, no); taking medication (number); and chronic disease (“Do you have a disease [presence of hypertension, stroke, heart disease, diabetes, hyperlipidemia, digestive disease, respiratory disease, urological diseases, and cancer]?”: yes, no). Comorbidity scores were calculated from the data obtained on nine comorbidity statuses. The summed value indicated a total score ranging from 0 (no comorbidity) to 9 (poor status). The previous week’s physical activity and sitting time per day were evaluated using the International Physical Activity Questionnaire-Short Form. Body mass index was calculated as self-reported body weight divided by the height squared (kg/m^2^).

**Supplemental Table 1**. Characteristics of participants in baseline and additional surveys and accelerometer study in the Kyoto-Kameoka Study

|  | Only first survey | |  | First and second survey | |  | Accelerometer study | |
| --- | --- | --- | --- | --- | --- | --- | --- | --- |
|  | All participants (*n* = 13294) | |  | All participants (*n* = 8319) | |  | Included participants (*n* = 4065) | |
| Age [years] ^a^ | 74.5 | (6.9) |  | 73.6 | (6.2) |  | 72.2 | (5.1) |
| Women [n (%)] ^b^ | 7337 | (55.2) |  | 4412 | (53.0) |  | 1972 | (48.5) |
| PD ≥1000 people/km^2^ [n (%)] ^b^ | 5917 | (44.5) |  | 3814 | (45.8) |  | 1985 | (48.8) |
| Body mass index [kg/m^2^] ^a^ | 22.5 | (3.6) |  | 22.6 | (3.5) |  | 22.7 | (3.1) |
| Living alone [n (%)] ^b^ | 1695 | (12.8) |  | 966 | (11.6) |  | 455 | (11.2) |
| HSES [n (%)] ^b^ | 4228 | (31.8) |  | 2770 | (33.3) |  | 1396 | (34.3) |
| Sleep time [min/day] ^a^ | 412 | (94) |  | 404 | (82) |  | 400 | (78) |
| Sitting time [min/day] ^a^ | 336 | (237) |  | 311 | (218) |  | 300 | (197) |
| Education ≥13 y [n (%)] ^b^ | 2567 | (19.3) |  | 1745 | (21.0) |  | 915 | (22.5) |
| Current smoker [n (%)] ^b^ | 1397 | (10.5) |  | 867 | (10.4) |  | 408 | (10.0) |
| Alcohol drinker [n (%)] ^b^ | 8277 | (62.3) |  | 5470 | (65.8) |  | 2745 | (67.5) |
| Denture use [n (%)] ^b^ | 8418 | (63.3) |  | 5127 | (61.6) |  | 2263 | (55.7) |
| No medication [n (%)] ^b^ | 2604 | (19.6) |  | 1826 | (21.9) |  | 954 | (23.5) |
| No. of chronic diseases ^a,c^ | 0.96 | (1.00) |  | 0.95 | (0.97) |  | 0.92 | (0.95) |
| Frailty [n (%)] ^b^ | 5321 | (44.4) |  | 2768 | (36.9) |  | 1013 | (24.9) |
| Disability ^d^ |  |  |  |  |  |  |  |  |
| n | 12443 | |  | 8167 | |  | 4065 | |
| Event | 3046 | |  | 1536 | |  | 385 | |
| Person-years | 54562 | |  | 34313 | |  | 12855 | |
| Event/1000 PY [95%CI] | 55.8 | (53.9 to 57.8) |  | 44.8 | (42.6 to 47.1) |  | 30.0 | (27.1 to 33.1) |

CI, confidence interval; HSES, high socioeconomic status; PD, population density; PY, person-years. Missing values were supplemented using the multivariate imputation method in all participants (*n* = 13294): body mass index (*n* = 1039; 7.8%), smoking status (*n* = 702; 5.3%), alcohol drinker (*n* = 604; 4.5%), family structure (*n* = 1119; 8.4%), socioeconomic status (*n* = 730; 5.5%), educational attainment (*n* = 1895; 14.3%), sleep duration (*n* = 1037; 7.8%), sitting time (*n* = 1895; 14.2%), denture use (*n* = 405; 3.0%), medications (*n* = 1140; 8.6%), and frailty status (*n* = 1722; 13.0%). Body mass index was calculated as body weight (kg) divided by height squared (m^2^).

^a^ Continuous values are shown as mean (standard deviation).

^b^ Categorical values are shown as number (percentage).

^c^ From the data obtained on disease status (including the presence of hypertension, stroke, heart disease, diabetes, hyperlipidemia, digestive disease, respiratory disease, urological diseases, and cancer), the comorbidity scores were summed to obtain a total score ranging from 0 (no comorbidity) to 9 (poor status).

^d^ Disability was calculated using data from July 30, 2011, in the first survey, February 14, 2012, in the second survey, and April 1 to November 15, 2013, in the accelerometer study, to November 30, 2016. Disability risk is shown as a rate (95% CI) per 1000 person-years.

**Supplemental Table 2**. Results of sensitivity analysis for the relationship between daily step count and risk of disability after excluding participants with an event in the first year of follow-up

| Quartile  (mean step) | *n* | Event | PY | Event/1000 PY | | Model 1^a^ | | Model 2^b^ | |
| --- | --- | --- | --- | --- | --- | --- | --- | --- | --- |
|  |  |  |  | Rate | 95% CI | SHR | 95% CI | SHR | 95% CI |
| **Total** |  | | | | | | | | |
| Q1 (1815) | 959 | 129 | 2999 | 43.0 | (36.2 to 51.1) | 1.00 | (Ref) | 1.00 | (Ref) |
| Q2 (3071) | 987 | 72 | 3182 | 22.6 | (18.0 to 28.5) | 0.73 | (0.54 to 0.99) | 0.79 | (0.58 to 1.07) |
| Q3 (4495) | 1000 | 48 | 3285 | 14.6 | (11.0 to 19.4) | 0.56 | (0.39 to 0.79) | 0.64 | (0.45 to 0.91) |
| Q4 (7550) | 1005 | 34 | 3323 | 10.2 | (7.3 to 14.3) | 0.48 | (0.33 to 0.72) | 0.61 | (0.41 to 0.91) |
| **Frailty**^c^ |  | | | | | | | | |
| Q1 (1553) | 227 | 57 | 678 | 84.0 | (64.8 to 108.9) | 1.00 | (Ref) | 1.00 | (Ref) |
| Q2 (2543) | 239 | 41 | 738 | 55.5 | (40.9 to 75.4) | 0.74 | (0.50 to 1.11) | 0.72 | (0.47 to 1.10) |
| Q3 (3770) | 244 | 30 | 781 | 38.4 | (26.9 to 54.9) | 0.63 | (0.40 to 0.98) | 0.59 | (0.35 to 0.98) |
| Q4 (6536) | 250 | 16 | 816 | 19.6 | (12.0 to 32.0) | 0.39 | (0.22 to 0.69) | 0.41 | (0.23 to 0.76) |
| **Non-frailty**^c^ |  | | | | | | | | |
| Q1 (1945) | 735 | 62 | 2347 | 26.4 | (20.6 to 33.9) | 1.00 | (Ref) | 1.00 | (Ref) |
| Q2 (3271) | 745 | 33 | 2425 | 13.6 | (9.7 to 19.1) | 0.74 | (0.48 to 1.17) | 0.70 | (0.44 to 1.11) |
| Q3 (4727) | 757 | 26 | 2503 | 10.4 | (7.1 to 15.3) | 0.64 | (0.40 to 1.04) | 0.63 | (0.39 to 1.04) |
| Q4 (7826) | 754 | 18 | 2502 | 7.2 | (4.5 to 11.4) | 0.55 | (0.32 to 0.95) | 0.58 | (0.33 to 1.03) |
| *p for between groups* |  |  |  |  |  | <0.001 | | <0.001 | |

CI, confidence interval; Q, quartile; Ref, reference; SHR, subdistribution hazard ratio; PY, person-years. Q1 through Q4 include daily step counts of <2477, 2478–3691, 3692–5419, and ≥5420 steps, respectively, in the total participants; <2079, 2080–3079, 3085–4565, and ≥4587 steps, respectively, in frail individuals; and <2643, 2644–3937, 3938–5682, and ≥5683 steps, respectively, in non-frail individuals. The step counts are expressed in these quartiles (mean values).

^a^ Model 1: Adjusted for age, sex, population density, and season of wear

^b^ Model 2: Adjusted for Model 1 variables and body mass index, smoking status, alcohol consumption status, family structure, educational attainment, economic status, sleep duration, sitting time, denture use, medication use, number of chronic diseases, and/or frailty status.

^c^ Variables (frailty) used for subgroup analysis were excluded from the adjustment for covariate variables in the model.

**Supplemental Table 3**. Results of sensitivity analysis for the relationship between daily step count and risk of disability using a multiple imputation method for missing values of covariates

| Quartile  (mean step) | *n* | Event | PY | Event/1000 PY | | Model 1^a^ | | Model 2^b^ | |
| --- | --- | --- | --- | --- | --- | --- | --- | --- | --- |
|  |  |  |  | Rate | 95% CI | SHR | 95% CI | SHR | 95% CI |
| **Total** |  | | | | | | | | |
| Q1 (1815) | 1018 | 182 | 3031 | 60.0 | (51.9 to 69.4) | 1.00 | (Ref) | 1.00 | (Ref) |
| Q2 (3071) | 1015 | 98 | 3199 | 30.6 | (25.1 to 37.3) | 0.73 | (0.56 to 0.93) | 0.77 | (0.60 to 1.00) |
| Q3 (4495) | 1016 | 62 | 3294 | 18.8 | (14.7 to 24.1) | 0.53 | (0.39 to 0.71) | 0.61 | (0.45 to 0.83) |
| Q4 (7550) | 1016 | 43 | 3330 | 12.9 | (9.6 to 17.4) | 0.45 | (0.32 to 0.63) | 0.56 | (0.39 to 0.80) |
| **Frailty**^c^ |  | | | | | | | | |
| Q1 (1553) | 254 | 83 | 693 | 119.7 | (96.6 to 148.5) | 1.00 | (Ref) | 1.00 | (Ref) |
| Q2 (2543) | 253 | 55 | 745 | 73.8 | (56.7 to 96.1) | 0.72 | (0.51 to 1.01) | 0.68 | (0.48 to 0.99) |
| Q3 (3770) | 253 | 39 | 787 | 49.6 | (36.2 to 67.8) | 0.59 | (0.40 to 0.88) | 0.60 | (0.39 to 0.92) |
| Q4 (6536) | 253 | 19 | 818 | 23.2 | (14.8 to 36.4) | 0.34 | (0.20 to 0.57) | 0.37 | (0.22 to 0.64) |
| **Non-frailty**^c^ |  | | | | | | | | |
| Q1 (1945) | 763 | 85 | 2363 | 36.0 | (29.1 to 44.5) | 1.00 | (Ref) | 1.00 | (Ref) |
| Q2 (3271) | 763 | 48 | 2436 | 19.7 | (14.8 to 26.1) | 0.80 | (0.55 to 1.16) | 0.79 | (0.54 to 1.15) |
| Q3 (4727) | 763 | 31 | 2506 | 12.4 | (8.7 to 17.6) | 0.57 | (0.37 to 0.88) | 0.57 | (0.37 to 0.89) |
| Q4 (7826) | 763 | 25 | 2507 | 10.0 | (6.7 to 14.8) | 0.57 | (0.35 to 0.91) | 0.61 | (0.37 to 1.01) |
| *p for between groups* |  |  |  |  |  | <0.001 | | <0.001 | |

CI, confidence interval; Q, quartile; Ref, reference; SHR, subdistribution hazard ratio; PY, person-years. Q1 through Q4 include daily step counts of <2477, 2478–3691, 3692–5419, and ≥5420 steps, respectively, in the total participants; <2079, 2080–3079, 3085–4565, and ≥4587 steps, respectively, in frail individuals; and <2643, 2644–3937, 3938–5682, and ≥5683 steps, respectively, in non-frail individuals. The step counts are expressed in these quartiles (mean values).

^a^ Model 1: Adjusted for age, sex, population density, and season of wear

^b^ Model 2: Adjusted for Model 1 variables and body mass index, smoking status, alcohol consumption status, family structure, educational attainment, economic status, sleep duration, sitting time, denture use, medication use, number of chronic diseases, and/or frailty status.

^c^ Variables (frailty) used for subgroup analysis were excluded from the adjustment for covariate variables in the model.

**Supplemental Table 4**. Results of sensitivity analysis for the relationship between daily step count and risk of disability after adjusting for each of the chronic diseases

| Quartile  (mean step) | *n* | Event | PY | Event/1000 PY | | Model 1^a^ | | Model 2^b^ | |
| --- | --- | --- | --- | --- | --- | --- | --- | --- | --- |
|  |  |  |  | Rate | 95% CI | SHR | 95% CI | SHR | 95% CI |
| **Total** |  | | | | | | | | |
| Q1 (1815) | 1018 | 182 | 3031 | 60.0 | (51.9 to 69.4) | 1.00 | (Ref) | 1.00 | (Ref) |
| Q2 (3071) | 1015 | 98 | 3199 | 30.6 | (25.1 to 37.3) | 0.73 | (0.56 to 0.93) | 0.74 | (0.57 to 0.96) |
| Q3 (4495) | 1016 | 62 | 3294 | 18.8 | (14.7 to 24.1) | 0.53 | (0.39 to 0.71) | 0.59 | (0.43 to 0.81) |
| Q4 (7550) | 1016 | 43 | 3330 | 12.9 | (9.6 to 17.4) | 0.45 | (0.32 to 0.63) | 0.55 | (0.38 to 0.79) |
| **Frailty**^c^ |  | | | | | | | | |
| Q1 (1553) | 254 | 83 | 693 | 119.7 | (96.6 to 148.5) | 1.00 | (Ref) | 1.00 | (Ref) |
| Q2 (2543) | 253 | 55 | 745 | 73.8 | (56.7 to 96.1) | 0.72 | (0.51 to 1.01) | 0.64 | (0.43 to 0.95) |
| Q3 (3770) | 253 | 39 | 787 | 49.6 | (36.2 to 67.8) | 0.59 | (0.40 to 0.88) | 0.56 | (0.36 to 0.88) |
| Q4 (6536) | 253 | 19 | 818 | 23.2 | (14.8 to 36.4) | 0.34 | (0.20 to 0.57) | 0.35 | (0.20 to 0.61) |
| **Non-frailty**^c^ |  | | | | | | | | |
| Q1 (1945) | 763 | 85 | 2363 | 36.0 | (29.1 to 44.5) | 1.00 | (Ref) | 1.00 | (Ref) |
| Q2 (3271) | 763 | 48 | 2436 | 19.7 | (14.8 to 26.1) | 0.80 | (0.55 to 1.16) | 0.77 | (0.53 to 1.14) |
| Q3 (4727) | 763 | 31 | 2506 | 12.4 | (8.7 to 17.6) | 0.57 | (0.37 to 0.88) | 0.56 | (0.36 to 0.88) |
| Q4 (7826) | 763 | 25 | 2507 | 10.0 | (6.7 to 14.8) | 0.57 | (0.35 to 0.91) | 0.62 | (0.37 to 1.03) |
| *p for between groups* |  |  |  |  |  | <0.001 | | <0.001 | |

CI, confidence interval; Q, quartile; Ref, reference; SHR, subdistribution hazard ratio; PY, person-years. Q1 through Q4 include daily step counts of <2477, 2478–3691, 3692–5419, and ≥5420 steps, respectively, in the total participants; <2079, 2080–3079, 3085–4565, and ≥4587 steps, respectively, in frail individuals; and <2643, 2644–3937, 3938–5682, and ≥5683 steps, respectively, in non-frail individuals. The step counts are expressed in these quartiles (mean values).

^a^ Model 1: Adjusted for age, sex, population density, and season of wear

^b^ Model 2: Adjusted for Model 1 variables and body mass index, smoking status, alcohol consumption status, family structure, educational attainment, economic status, sleep duration, sitting time, denture use, medication use, history of hypertension, stroke, heart disease, diabetes, hyperlipidemia, digestive disease, respiratory disease, urological diseases, and cancer.

^c^ Variables (frailty) used for subgroup analysis were excluded from the adjustment for covariate variables in the model.

**Supplemental Table 5**. Results of sensitivity analysis for the association of the interaction between daily step count and frailty with disability after excluding participants with an event in the first year of follow-up

|  | *n* | Event | PY | Event/1000 PY | | Model 1^a^ | | Model 2^b^ | |
| --- | --- | --- | --- | --- | --- | --- | --- | --- | --- |
|  |  |  |  | Rate | 95% CI | SHR | 95% CI | SHR | 95% CI |
| **SC×Frail** |  | | | | | | | | |
| HSC/Non-frail | 995 | 30 | 3302 | 9.1 | (6.4 to 13.0) | 1.00 | (Ref) | 1.00 | (Ref) |
| HSC/Frail | 203 | 13 | 664 | 19.6 | (11.4 to 33.7) | 1.74 | (0.90 to 3.36) | 1.39 | (0.69 to 2.80) |
| LSC/Non-frail | 1996 | 109 | 6474 | 16.8 | (14.0 to 20.3) | 1.28 | (0.85 to 1.93) | 1.22 | (0.80 to 1.86) |
| LSC/Frail | 757 | 131 | 2349 | 55.8 | (47.0 to 66.2) | 3.07 | (2.03 to 4.65) | 2.35 | (1.52 to 3.64) |
| *Interaction* |  |  |  |  |  |  |  |  |  |
| RERI^c^ |  |  |  |  |  | 1.05 | (0.16 to 1.94) | 0.74 | (0.04 to 1.83) |
| *p*-value |  |  |  |  |  | 0.007 | | 0.044 | |
| Multiplicative |  |  |  |  | | 1.37 | (0.68 to 2.79) | 1.39 | (0.66 to 2.93) |
| *p*-value |  |  |  |  |  | 0.380 | | 0.388 | |
| **Step counts** |  |  |  |  |  |  |  |  |  |
| ≥5000 steps | 1198 | 43 | 3967 | 10.8 | (8.0 to 14.6) | 1.00 | (Ref) | 1.00 | (Ref) |
| <5000 steps | 2753 | 240 | 8823 | 27.2 | (24.0 to 30.9) | 1.44 | (1.03 to 2.01) | 1.37 | (0.97 to 1.93) |
| **Frailty status** |  |  |  |  |  |  |  |  |  |
| Non-frail | 2991 | 139 | 9777 | 14.2 | (12.0 to 16.8) | 1.00 | (Ref) | 1.00 | (Ref) |
| Frail | 960 | 144 | 3013 | 47.8 | (40.6 to 56.3) | 2.29 | (1.79 to 2.92) | 1.84 | (1.42 to 2.39) |

CI, confidence interval; SHR, subdistribution hazard ratio; HSC, high step counts; LSC, low step counts; PY, person years; Ref, reference; RERI, Relative Excess Risk due to Interaction; SC, step counts

^a^ Model 1: Adjusted for age, sex, population density, and season of wear

^b^ Model 2: Adjusted for Model 1 variables and body mass index, smoking status, alcohol consumption status, family structure, educational attainment, economic status, sleep duration, sitting time, denture use, medication use, number of chronic diseases, and/or frailty status.

^c^ The additive interaction was calculated as the RERI using the following equation: RERI = (SHR [LSC/Frail] − 1) − (SHR [LSC/Non-frail] + SHR [HSC/Frail] − 2). The values are shown as RERI (95% CI). It is significant (p < 0.05) if the 95% CI of the RERI is not above 0.

**Supplemental Table 6**. Results of sensitivity analysis for the association of the interaction between daily step count and frailty with disability using a multiple imputation method for missing values of covariates

|  | *n* | Event | PY | Event/1000 PY | | Model 1^a^ | | Model 2^b^ | |
| --- | --- | --- | --- | --- | --- | --- | --- | --- | --- |
|  |  |  |  | Rate | 95% CI | SHR | 95% CI | SHR | 95% CI |
| **SC×Frail** |  | | | | | | | | |
| HSC/Non-frail | 1007 | 39 | 3310 | 11.8 | (8.6 to 16.1) | 1.00 | (Ref) | 1.00 | (Ref) |
| HSC/Frail | 206 | 16 | 666 | 24.0 | (14.7 to 39.2) | 1.35 | (0.94 to 1.93) | 1.40 | (0.76 to 2.58) |
| LSC/Non-frail | 2045 | 150 | 6502 | 23.1 | (19.7 to 27.1) | 1.64 | (0.91 to 2.96) | 1.28 | (0.89 to 1.84) |
| LSC/Frail | 807 | 180 | 2377 | 75.7 | (65.4 to 87.7) | 3.12 | (2.17 to 4.49) | 2.47 | (1.69 to 3.60) |
| *Interaction* |  |  |  |  |  |  |  |  |  |
| RERI^c^ |  |  |  |  |  | 1.13 | (0.35 to 1.91) | 0.79 | (0.11 to 1.47) |
| *p*-value |  |  |  |  |  | 0.001 | | 0.016 | |
| Multiplicative |  |  |  |  |  | 1.41 | (0.75 to 2.65) | 1.38 | (0.72 to 2.64) |
| *p*-value |  |  |  |  |  | 0.285 | | 0.326 | |
| **Step counts** |  |  |  |  |  |  |  |  |  |
| ≥5000 steps | 1213 | 55 | 3976 | 13.8 | (10.6 to 18.0) | 1.00 | (Ref) | 1.00 | (Ref) |
| <5000 steps | 2852 | 330 | 8879 | 37.2 | (33.4 to 41.4) | 1.67 | (1.24 to 2.23) | 1.43 | (1.06 to 1.93) |
| **Frailty status** |  |  |  |  |  |  |  |  |  |
| Non-frail | 3052 | 189 | 9812 | 19.3 | (16.7 to 22.2) | 1.00 | (Ref) | 1.00 | (Ref) |
| Frail | 1013 | 196 | 3043 | 64.4 | (56.0 to 74.1) | 2.20 | (1.78 to 2.71) | 1.85 | (1.49 to 2.30) |

CI, confidence interval; SHR, subdistribution hazard ratio; HSC, high step counts; LSC, low step counts; PY, person years; Ref, reference; RERI, Relative Excess Risk due to Interaction; SC, step counts

^a^ Model 1: Adjusted for age, sex, population density, and season of wear

^b^ Model 2: Adjusted for Model 1 variables and body mass index, smoking status, alcohol consumption status, family structure, educational attainment, economic status, sleep duration, sitting time, denture use, medication use, number of chronic diseases, and/or frailty status.

^c^ The additive interaction was calculated as the RERI using the following equation: RERI = (SHR [LSC/Frail] − 1) − (SHR [LSC/Non-frail] + SHR [HSC/Frail] − 2). The values are shown as RERI (95% CI). It is significant (p < 0.05) if the 95% CI of the RERI is not above 0.

**Supplemental Table 7**. Results of sensitivity analysis for the association of the interaction between daily step count and frailty with disability after adjusting for each of the chronic diseases

|  | *n* | Event | PY | Event/1000 PY | | Model 1^a^ | | Model 2^b^ | |
| --- | --- | --- | --- | --- | --- | --- | --- | --- | --- |
|  |  |  |  | Rate | 95% CI | SHR | 95% CI | SHR | 95% CI |
| **SC×Frail** |  | | | | | | | | |
| HSC/Non-frail | 1007 | 39 | 3310 | 11.8 | (8.6 to 16.1) | 1.00 | (Ref) | 1.00 | (Ref) |
| HSC/Frail | 206 | 16 | 666 | 24.0 | (14.7 to 39.2) | 1.35 | (0.94 to 1.93) | 1.26 | (0.67 to 2.36) |
| LSC/Non-frail | 2045 | 150 | 6502 | 23.1 | (19.7 to 27.1) | 1.64 | (0.91 to 2.96) | 1.25 | (0.87 to 1.81) |
| LSC/Frail | 807 | 180 | 2377 | 75.7 | (65.4 to 87.7) | 3.12 | (2.17 to 4.49) | 2.35 | (1.61 to 3.45) |
| *Interaction* |  |  |  |  |  |  |  |  |  |
| RERI^c^ |  |  |  |  |  | 1.13 | (0.35 to 1.91) | 0.85 | (0.17 to 1.54) |
| *p*-value |  |  |  |  |  | 0.001 | | 0.012 | |
| Multiplicative |  |  |  |  | | 1.41 | (0.75 to 2.65) | 1.50 | (0.77 to 2.90) |
| *p*-value |  |  |  |  |  | 0.285 | | 0.231 | |
| **Step counts** |  |  |  |  |  |  |  |  |  |
| ≥5000 steps | 1213 | 55 | 3976 | 13.8 | (10.6 to 18.0) | 1.00 | (Ref) | 1.00 | (Ref) |
| <5000 steps | 2852 | 330 | 8879 | 37.2 | (33.4 to 41.4) | 1.67 | (1.24 to 2.23) | 1.52 | (1.12 to 2.06) |
| **Frailty status** |  |  |  |  |  |  |  |  |  |
| Non-frail | 3052 | 189 | 9812 | 19.3 | (16.7 to 22.2) | 1.00 | (Ref) | 1.00 | (Ref) |
| Frail | 1013 | 196 | 3043 | 64.4 | (56.0 to 74.1) | 2.20 | (1.78 to 2.71) | 1.82 | (1.46 to 2.28) |

CI, confidence interval; SHR, subdistribution hazard ratio; HSC, high step counts; LSC, low step counts; PY, person years; Ref, reference; RERI, Relative Excess Risk due to Interaction; SC, step counts

^a^ Model 1: Adjusted for age, sex, population density, and season of wear

^b^ Model 2: Adjusted for Model 1 variables and body mass index, smoking status, alcohol consumption status, family structure, educational attainment, economic status, sleep duration, sitting time, denture use, medication use, history of hypertension, stroke, heart disease, diabetes, hyperlipidemia, digestive disease, respiratory disease, urological diseases, and cancer, and/or frailty status.

^c^ The additive interaction was calculated as the RERI using the following equation: RERI = (SHR [LSC/Frail] − 1) − (SHR [LSC/Non-frail] + SHR [HSC/Frail] − 2). The values are shown as RERI (95% CI). It is significant (p < 0.05) if the 95% CI of the RERI is not above 0.
